# Measuring attitudes toward artificial intelligence: validation of the Chinese ATTARI-12 and invariance across genders and academic disciplines

**DOI:** 10.3389/fpsyg.2026.1836060

**Published:** 2026-05-15

**Authors:** Ping Li, Yong-Yee Chong, Wei Man, Hai Li

**Affiliations:** 1 Department of Psychiatry, The Second People's Hospital of Neijiang, Neijiang, China; 2 Faculty of Sports and Exercise Science, Universiti Malaya, Kuala Lumpur, Malaysia; 3 School of Foreign Languages, Neijiang Normal University, Neijiang, China; 4 College of Sport, Neijiang Normal University, Neijiang, China

**Keywords:** artificial intelligence, attitudes, Chinese undergraduates, confirmatory factor analysis, measurement invariance, psychometric validation

## Abstract

**Introduction:**

Understanding university students’ attitudes toward artificial intelligence (AI) is essential for its ethical and effective integration into higher education. However, validated tools tailored to the Chinese context are limited.

**Methods:**

This study translated and validated the Chinese version of the Attitudes toward Artificial Intelligence Scale (ATTARI-12) using Brislin’s translation procedure. A cross-sectional online survey was conducted among 920 undergraduate students. The dataset was split for exploratory and confirmatory factor analyses (EFA and CFA), followed by internal consistency assessment and multi-group CFA to test measurement invariance across gender and academic disciplines. Descriptive statistics and group comparisons were performed.

**Results:**

The EFA and CFA supported a stable two-factor structure reflecting positive and negative attitudes, with acceptable reliability (*α* = 0.79; *ω* = 0.82). The scale showed full scalar invariance across genders and partial invariance across academic disciplines. Descriptive results indicated generally favorable student attitudes toward AI, with no significant differences across genders and disciplines.

**Conclusion:**

The Chinese ATTARI-12 demonstrates sound psychometric properties and is suitable for assessing AI-related attitudes among undergraduate students.

## Introduction

### Background

Over the past decade, the application of artificial intelligence (AI) has expanded rapidly across various industries, and the education sector is no exception. The emergence of generative AI technologies such as ChatGPT, Google Gemini, and other large language models has significantly accelerated the integration of AI into higher education (McGrath et al., 2025). Governments, universities, and industry stakeholders worldwide are actively promoting the adoption of AI to enhance teaching quality, support personalized learning, and improve institutional efficiency. Governments are playing a leading role in driving AI integration across education systems. In the United States, the Department of Education’s 2023 report, *Artificial Intelligence and the Future of Teaching and Learning*, outlines strategies for the effective integration of AI into education (U.S. Department of Education, 2023), while Ohio has become one of the first states to mandate that all school districts establish AI usage policies (Neese, 2025). In China, the government announced a major education reform initiative to integrate AI into teaching, textbooks, and curricula at all levels of education (Reuters, 2025). Internationally, the United Nations Educational, Scientific and Cultural Organization introduced AI competency frameworks in 2024 to guide the integration of AI knowledge, ethics, and pedagogy among students and teachers (United Nations Educational, 2024). Higher education institutions worldwide are also actively launching undergraduate and postgraduate programs focused on AI (Cheng, 2024; Saklaki and Gardikiotis, 2024). AI is currently widely applied in areas such as adaptive learning platforms, automated assessment systems, academic writing tools, and virtual lab simulations (Saklaki and Gardikiotis, 2024). For university students, AI tools have become an integral part of their daily academic life, offering strong support for coursework, language learning, and career planning (Laato et al., 2023; Baskara et al., 2024). Additionally, industry stakeholders such as the Brookings Global Task Force advocated the use of generative AI to support global educational transformation (Brookings, 2024).

However, concerns have emerged amid this growing adoption. Researchers are raising concerns about the use of AI in higher education, particularly regarding students’ overreliance on AI, threats to academic integrity, and educators’ preparedness. Studies have shown that university students frequently use AI tools such as ChatGPT to assist with writing and completing assignments, which may lead to overdependence and diminish critical thinking and originality (Garil et al., 2024; Barrientos et al., 2024). A study conducted in Kazakhstan reported that nearly all surveyed university students hoped that institutions would introduce clear policies to ensure that AI use would not undermine academic integrity (Askarkyzy and Zhunusbekova, 2024). In Canada, researchers have found that most universities lack explicit guidelines for integrating AI into academic integrity frameworks, leaving both students and staff uncertain about their appropriate use (Marcel and Kang, 2024). Furthermore, researchers have suggested that the gap between institutional expectations and educator readiness continues to hinder the pedagogically sound and ethically responsible use of AI in university teaching (Striepe et al., 2021). Given this context, understanding how university students perceive and respond to AI technology is essential. As both active users and primary stakeholders in AI-driven educational reforms, their attitudes may influence their levels of engagement, ethical judgment, and overall academic outcomes. Gaining insight into these attitudes is therefore critical for the responsible and effective integration of AI in higher education.

To date, studies on university students’ attitudes toward AI have employed a variety of approaches, including qualitative interviews, self-developed questionnaires, and general technology acceptance models (Tien, 2024; Alzahrani, 2023; Ma et al., 2024). However, many of these instruments were not specifically designed to assess attitudes toward AI and often fail to capture the multidimensional nature of attitudinal constructs. Widely used frameworks such as the Technology Acceptance Model (TAM) and the Unified Theory of Acceptance and Use of Technology (UTAUT) were originally developed to explain individuals’ adoption and usage of general technologies. These models primarily emphasize perceived usefulness, ease of use, and behavioral intention. While effective for predicting technology adoption, they offer a relatively narrow perspective when applied to AI, as they do not explicitly account for affective responses, ethical concerns, or broader evaluative beliefs about AI as a transformative and potentially disruptive technology (Alzahrani, 2023; Ma et al., 2024; Osman et al., 2024). This limitation has been acknowledged by researchers; Alzahrani (2023) noted that traditional acceptance models often fail to account for factors specific to AI. Recent research further suggests that attitudes toward AI should be conceptualized within a broader theoretical framework that integrates technology acceptance, trust, and socio-cognitive evaluation processes. Empirical evidence indicates that factors such as perceived usefulness, ease of use, social influence, self-efficacy, and perceived risk jointly shape students’ acceptance of AI technologies (Nurtanto et al., 2025; Hamkah et al., 2025). However, attitudes toward AI extend beyond behavioral intention. They also involve evaluative judgments regarding the reliability, autonomy, and potential risks of AI systems. In this regard, trust in AI has been identified as a critical determinant influencing users’ willingness to rely on algorithmic systems (Hamkah et al., 2025; Sutrisno et al., 2025). Moreover, recent studies in educational contexts have shown that AI-related attitudes are closely associated with learning engagement, satisfaction, and perceived effectiveness of AI-supported systems (Feladi et al., 2025; Özbay, 2026). These findings suggest that AI attitudes are inherently multidimensional, incorporating affective, cognitive, and evaluative components. Similarly, in studies conducted in the Chinese context, most surveys assessing university students’ attitudes toward AI primarily rely on general technology acceptance models, while few studies use scales specifically designed to measure attitudes toward AI (Ma et al., 2024; Huang et al., 2024; Li et al., 2025). The Attitudes toward Artificial Intelligence Scale (ATTARI-12) is a promising solution (Stein et al., 2024). Grounded in the tripartite model of attitudes, cognitive, affective, and behavioral, which was specifically developed to assess general evaluations of AI rather than technology adoption or usage. This distinguishes ATTARI-12 from traditional acceptance-based frameworks such as TAM and UTAUT, which primarily focus on behavioral intention and system use.

Attitudes toward AI may not be uniformly constructed across different academic disciplines. Students from STEM (Science, Technology, Engineering, and Mathematics) backgrounds may be more likely to engage with AI as a technical or computational system, whereas students in the humanities and social sciences may approach AI through ethical, societal, or critical perspectives (Ma et al., 2024; Li et al., 2025). These potential differences do not necessarily imply actual group disparities but highlight the need to examine whether the construct is interpreted equivalently across disciplines. In this context, ATTARI-12 is particularly suitable as it captures broad attitudinal orientations, including emotional and value-based evaluations (Briesemeister et al., 2012; Rocklage and Fazio, 2016), that are not restricted to specific usage scenarios.

From a measurement perspective, these potential differences highlight the importance of examining whether the construct is interpreted equivalently across disciplinary groups. Therefore, testing measurement invariance across academic disciplines is theoretically motivated, as it evaluates the structural stability of AI-related attitudes across distinct knowledge domains.

The scale has shown good psychometric properties and cross-cultural applicability. As such, it offers a valuable foundation for examining how Chinese university students perceive AI in their current technological and educational climate (Stein et al., 2024).

### Aim

Given the need for a psychometrically sound tool to assess AI-related attitudes in the Chinese higher education context, this study was guided by two hypotheses:

*Hypothesis 1*: The Chinese version of the ATTARI-12 will demonstrate acceptable validity, reliability, and measurement invariance across gender and academic disciplines.

*Hypothesis 2*: While overall student attitudes toward AI are generally positive, significant differences may exist between genders and academic disciplines in terms of attitudinal patterns.

## Materials and methods

### Translation and content validity

This study adopted Brislin’s standard cross-cultural adaptation procedure, including forward translation, back translation, and expert panel review, to ensure semantic and conceptual equivalence of the Chinese version of the ATTARI-12 (Qin et al., 2025; Xu et al., 2019). The ATTARI-12 scale was originally developed and validated by Stein, Messingschlager, Gnambs, Hutmacher and Appel (Stein et al., 2024) and is publicly available via open access^1^. Initially, two bilingual researchers with expertise in AI independently conducted forward translations of the original instrument into Chinese. Discrepancies between the two versions were resolved through consensus-seeking meetings involving the research team, with a focus on achieving semantic, conceptual, and cultural equivalence. Subsequently, two independent bilingual translators, blinded to the original scale and without AI backgrounds, performed the back-translation. To ensure accuracy, the project leader compared the back-translated versions item by item with the original English scale. Any inconsistencies were iteratively refined until full equivalence was reached. To ensure face validity and comprehensibility, a pilot test was conducted with five native Chinese undergraduate students (from psychology and AI backgrounds). They evaluated the items for clarity, wording, and interpretability. Finally, an expert panel reviewed the synthesized version to confirm content validity, resulting in the final Chinese version of the ATTARI-12.

In this study, the I-CVI and S-CVI (Cai et al., 2024) were used to assess the content validity of the Chinese version of ATTARI-12. Seven academic researchers with backgrounds in AI and psychology were invited to participate in the evaluation. They used a four-point Likert scale to rate the relevance (1 = not relevant, 4 = highly relevant) and clarity (1 = not clear, 4 = highly clear) of each item. The I-CVI was calculated as the proportion of experts rating the item as 4, and the S-CVI was computed as the average of the I-CVI scores across all 12 items of ATTARI-12 (Cai et al., 2024; Polit et al., 2007).

### Participants and procedure

This study employed a single-time data collection method (Ng, 2013). The total sample was randomly divided into two approximately equal subsamples for cross-validation between EFA and CFA (Kyriazos, 2018). The required sample size was determined based on three analytical requirements: EFA, CFA, and group comparisons. For EFA, an item-to-sample ratio of 20:1 was adopted for the ATTARI-12 scale (Xu et al., 2019; Sapnas and Zeller, 2002), indicating a minimum of 240 participants. The total sample consisted of at least 480 participants, split into two subsamples (one for EFA and one for CFA, each with *n* = 240). A CFA-specific power analysis (specifying a null hypothesis RMSEA of 0.05 and an alternative RMSEA of 0.08, with df = 48, *α* = 0.01, and power = 0.90; conducted in R 4.5.0) indicated that a minimum of 153 participants per subsample was required. This requirement was well satisfied by the 240-per-subsample criterion, in line with recommended practices for structural equation modeling (Kim, 2016).

Additionally, an ANOVA-based power analysis (*f* = 0.25, *k* = 4, *α* = 0.01, power = 0.90; conducted in R 4.5.0) indicated that a minimum of approximately 79 participants per group (total *n* = 316) was required to detect moderate group differences across gender and academic discipline (Kim, 2016).

Therefore, the final target sample size was set at 565 to meet all analytical requirements while accounting for an estimated 15% invalid response rate. The final valid sample (*n* = 920) exceeded all required thresholds, supporting adequate statistical power for structural modeling and measurement invariance testing.

Data were collected using the online questionnaire platform “WJX”^2^. The participants were undergraduate students at a regional university in China. Before answering the questionnaire, participants were presented with a description of the study and an informed consent form that clearly outlined the study’s purpose, data anonymity protections, and their right to withdraw at any time.

In addition to the questionnaire items, participants provided basic demographic information, including age, gender, and academic discipline. For academic disciplines, a standardized list of undergraduate majors was embedded in the questionnaire for reference. Reported majors were subsequently classified by the research team into two domains—STEM and HSS (Humanities and Social Sciences) based on widely accepted categorizations in Chinese higher education (Wang et al., 2021). No other personal identifiable data was collected.

This study was reviewed and approved by the Ethics Review Committee of Neijiang Normal University (Approval No.: NJNU-KJC-2025004). All the procedures complied with the principles of the Declaration of Helsinki.

### Construct validity

#### Exploratory factor analysis (EFA)

The EFA was conducted on each of the two randomly divided subsamples to cross-validate the underlying factor structure of the Chinese version of ATTARI-12. The KMO index and Bartlett’s test of sphericity were used to assess sampling adequacy and the suitability of the data for factor analysis (Önder, 2023). Factor extraction was guided by the Kaiser criterion (eigenvalues > 1) and by visual inspection of the scree plot, and was supplemented by parallel analysis. Principal axis factoring with an oblimin rotation (oblique rotation method) was used. Oblique rotation was selected because the ATTARI-12’s latent factors were expected to be correlated. In attitude research, oblique rotation is generally preferred because the underlying constructions are conceptually related rather than orthogonal (Zhang et al., 2018; Welsh et al., 1990).

#### Confirmatory factor analysis (CFA)

The CFA was conducted on both subsamples to cross-validate the factor model based on the factor structures identified in the EFA results. Prior to performing the CFA, multivariate normality was tested using Mardia’s skewness and kurtosis statistics. Maximum Likelihood (ML) estimation was applied if the data met the assumption of normality; otherwise, Robust Maximum Likelihood (MLR) was used to enhance the accuracy of parameter estimates and the robustness of the model fit (Li, 2016; Zhong and Yuan, 2011).

Model fit was comprehensively assessed using multiple indices, including the chi-square to degrees of freedom ratio (*χ*^2^/df), CFI, TLI, RMSEA, and SRMR (Shi et al., 2021; Campos et al., 2014). No *post hoc* model modifications based on modification indices were applied, to preserve the theoretical structure of the original scale. Additionally, to further assess convergent validity, Composite reliability (CR) and average variance extracted (AVE) were calculated for each factor based on standardized factor loadings (Gebrekidan et al., 2024). To address potential common-method bias in self-reported data, Harman’s single-factor test was conducted using unrotated EFA (principal axis factoring) (Podsakoff et al., 2003).

### Internal consistency reliability

To assess the scale’s reliability, internal consistency was evaluated for the total sample prior to factor analysis. Cronbach’s alpha (*α*) was calculated for the overall scale to assess internal consistency, and McDonald’s omega (*ω*) was computed using principal axis factoring with oblimin rotation as a supplementary reliability estimate (Tavakol and Dennick, 2011). After the factor structure was established through the EFA, reliability was re-examined at the subscale level to verify the internal consistency of each factor (Zhang et al., 2020).

### Invariance test

To verify the measurement invariance of ATTARI-12 and its factor structure across gender and academic discipline groups, a MG-CFA was conducted using the total sample (Xu and Tracey, 2017). Following standard procedures for testing measurement invariance, three levels were assessed sequentially: configural, metric, and scalar invariance (Blanco-Canitrot et al., 2018).

### Descriptive statistics and group comparisons

If the Chinese version of the ATTARI-12 translated in this study is validated as having good psychometric properties, it can be used to examine college students’ attitudes toward AI using a valid sample.

First, descriptive statistics (e.g., mean, standard deviation, skewness, and kurtosis) were computed for each item and its corresponding factor to provide the response distribution and central tendencies.

Subsequently, group comparisons were performed to identify differences in attitudes across demographic variables (sex: male/female; academic discipline: STEM/HSS). The normality of factor scores was assessed using the Shapiro–Wilk test, and homogeneity of variances was assessed using Levene’s test. If the data meet the assumptions for parametric testing, for factorial comparisons (gender × academic discipline), ANOVA will be conducted if parametric assumptions are met (Kim, 2017). Otherwise, the nonparametric Scheirer–Ray–Hare test will be applied instead (Zhang et al., 2025). The significance level was set at *p* = 0.05.

### Data analysis

Content validity (I-CVI and S-CVI) was assessed based on expert ratings. I-CVI (≥0.78) was considered acceptable for individual items, and S-CVI (≥0.90) indicated high overall content validity (Polit et al., 2007). Reverse-coded items (Q2, Q4, Q7, Q8, Q10, and Q12) were recorded to ensure scoring consistency. After data cleaning, the total sample was randomly split into two equal subsets (1:1 ratio) for EFA and CFA to cross-validate.

The EFA was performed using principal axis factoring with oblimin rotation. Factor retention was based on eigenvalues >1, scree plot inspection, and theoretical interpretability (Korsch et al., 2022). Sampling adequacy was confirmed via KMO (>0.60) and Bartlett’s test (*p* < 0.05) (Pedrosa et al., 2016). CFA was conducted to validate the factor structure identified through the EFA. Model fit was evaluated using χ^2^/df (<3), CFI and TLI (≥0.90), RMSEA (≤0.08), and SRMR (≤0.08) (Campos et al., 2016). CR ≥ 0.70 and AVE ≥ 0.50 were considered indicative of adequate convergent validity (Pedrosa et al., 2016; Baharum et al., 2023).

Internal consistency was assessed using Cronbach’s alpha and McDonald’s omega (*α* and *ω* ≥ 0.70) (Tavakol and Dennick, 2011; Hayes and Coutts, 2020). Measurement invariance across gender and academic discipline was tested using multi-group CFA, with configural, metric, and scalar models evaluated via ΔCFI (<0.01), ΔRMSEA (<0.015), and ΔSRMR (<0.030 for metric, <0.015 for scalar) (Lin et al., 2017).

Data preprocessing (including data cleaning, re-coding of reverse-scored items, and random splitting of the dataset) and visualization were conducted in Python 3.13 (Python Software Foundation, 2023) using Visual Studio Code (Microsoft, 2023). All statistical analyses were performed using R version 4.5.0 (RC Team, 2025). Exploratory and confirmatory factor analyses were performed using the psych and *lavaan* packages, respectively. Reliability was assessed using *psych*, and measurement invariance was tested using the *semTools* package. Descriptive statistics and group comparisons were conducted using the *stats*, *car*, and *effsize* packages.

## Results

### Translation and content validity

Following the translation procedure, the Chinese version of the ATTARI-12 was developed and subsequently evaluated by seven domain experts. For transparency and reproducibility, the full set of ATTARI-12 items (Chinese and English versions) is provided in Supplementary Table S1.

Content validity indices indicated strong agreement among the expert raters. For both relevance and clarity, the Item-level Content Validity Index (I-CVI) values ranged from 0.857 to 1.000 across all the items. The Scale-level Content Validity Index (S-CVI) was 0.928 for relevance and 0.902 for clarity, indicating high content validity of the Chinese version of the ATTARI-12.

### Sample characteristics

A total of 1,115 questionnaires were collected between 1 May and 20 May 2025 via the online survey platform WJX (see Footnote 2). During data cleaning, invalid responses were excluded based on the following criteria: (1) completion time less than 90 s, (2) uniform response patterns (selecting the same option for all items), and (3) implausible age (<15 or >30 years). After screening, 920 valid responses (90.6%) were retained. The valid responses were randomly divided into two equal subsamples (1:1 ratio) for EFA and CFA. The descriptive characteristics of the total sample and subsamples are presented in Table 1.

**Table 1:** Descriptive characteristics of the participants (*n* = 920).

Sample	Gender	Academic discipline	Number	Age, Mean (SD)	Gender (%)	Academic discipline (%)
Total sample	Female	STEM	197	19.62 (0.97)	57.07	42.50
		HSS	328	19.52 (1.09)		
	Male	STEM	194	19.62 (1.17)	42.93	57.50
		HSS	201	20.04 (1.26)		
Subsample A	Female	STEM	89	19.60 (0.94)	28.70	20.54
		HSS	175	19.59 (1.09)		
	Male	STEM	100	19.53 (1.20)	21.30	29.46
		HSS	96	19.99 (1.22)		
Subsample B	Female	STEM	108	19.64 (0.99)	28.37	21.96
		HSS	153	19.43 (1.08)		
	Male	STEM	94	19.71 (1.14)	21.63	28.04
		HSS	105	20.09 (1.31)		

Percentages refer to the distribution within the total sample (*n* = 920).

The sample comprised undergraduate students aged 18–22 years, with a higher proportion of female students than male students and greater representation of students from HSS than from STEM. These distributions were consistent with the demographic and academic characteristics of the target population (Xu et al., 2023). The distribution of participants across gender and academic discipline groups was relatively balanced (male/female, STEM/HSS), ensuring adequate subgroup sample sizes for reliable multi-group CFA and invariance testing.

### Construct validity

#### EFA

The EFA was conducted separately on two randomly divided subsamples (Sample A and Sample B, *n* = 460 each) to cross-validate the factor structure of the Chinese version of the ATTARI-12. For Sample A, the Kaiser–Meyer–Olkin (KMO) measure of sampling adequacy was 0.83, and Bartlett’s test of sphericity was significant (*χ*^2^ = 1311.62, df = 66, *p* < 0.001), supporting the suitability of the data for factor analysis. For Sample B, the KMO was 0.85 and Bartlett’s test was also significant (*χ*^2^ = 1189.69, df = 66, *p* < 0.001), confirming the factorability of the correlation matrix.

**Figure 1:**
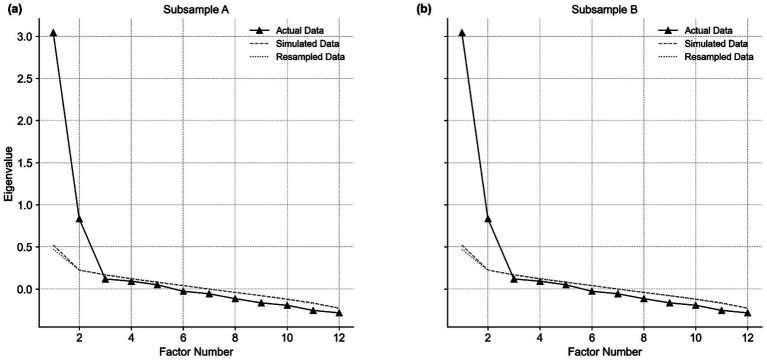
Parallel analysis scree plot. Panel **(a)** corresponds to Subsample A; **(b)** corresponds to Subsample B. The figure compares the actual data eigenvalues (solid black line with ▲ markers) with those from simulated random data (dashed gray line) and resampled data (dotted gray line).

**Figure 2:**
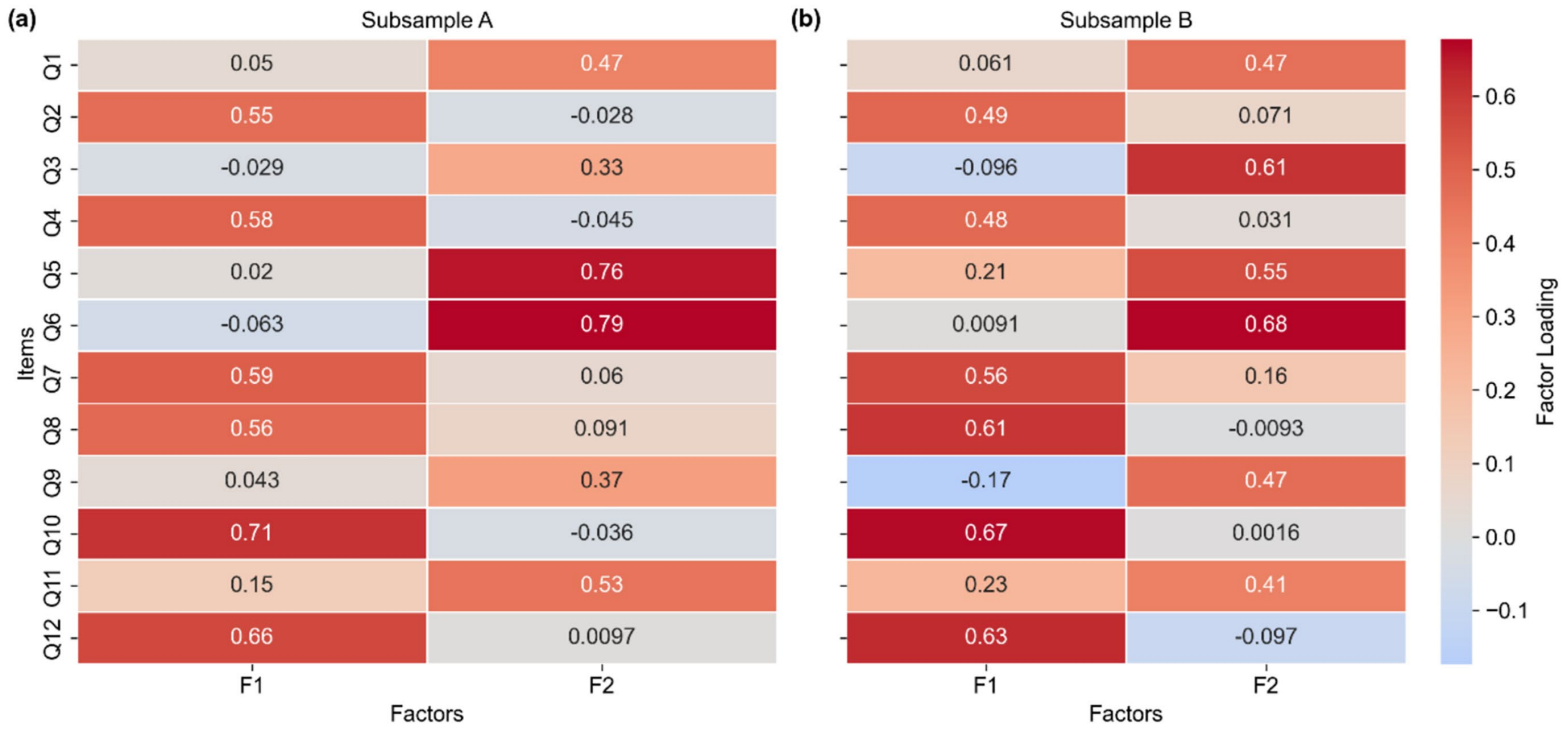
Heatmaps of factor loading. Panel **(a)** corresponds to Subsample A; **(b)** corresponds to Subsample B.

Principal axis factoring with oblimin rotation was applied to both samples. Parallel analysis consistently suggested a two-factor solution, which was further supported by eigenvalues >1 and a scree plot inspection (Figure 1). Heatmaps based on the factor-loading matrices were generated for both subsamples to provide a clearer visual representation of the item–factor relationships (Figure 2). The heatmaps demonstrate that each item loaded primarily onto one factor with negligible cross-loadings, and this structure remained consistent across both subsamples, supporting the factor clarity and structural stability of the ATTARI-12. Moreover, the heatmaps show that items Q2, Q4, Q7, Q8, Q10, and Q12 were predominantly loaded onto Factor 1 (F1; reverse-coded items), whereas items Q1, Q3, Q5, Q6, Q9, and Q11 were primarily associated with Factor 2 (F2; positively worded items) across both subsamples. The two retained factors explained 36% (Sample A: 19 and 17%) and 34% (Sample B: 19 and 15%) of the total variance, respectively. Moderate interfactor correlations (Sample A *r* = 0.44; Sample B: *r* = 0.47) justified the use of oblique rotation. Consistency in the factor structure across the two samples provided strong evidence for the stability of the identified dimensions. Overall, these results support the adequacy and structural stability of the two-factor solution.

#### CFA

The CFA was conducted on two subsamples (Sample A and B) to cross-validate the two-factor structure identified in the EFA. Both subsamples exhibited significant departures from multivariate normality (Sample A: skewness = 1857.4, kurtosis = 44.49, *p* < 0.001; Sample B: skewness = 1418.6, kurtosis = 29.17, *p* < 0.001). Therefore, MLR was applied.

**Figure 3:**
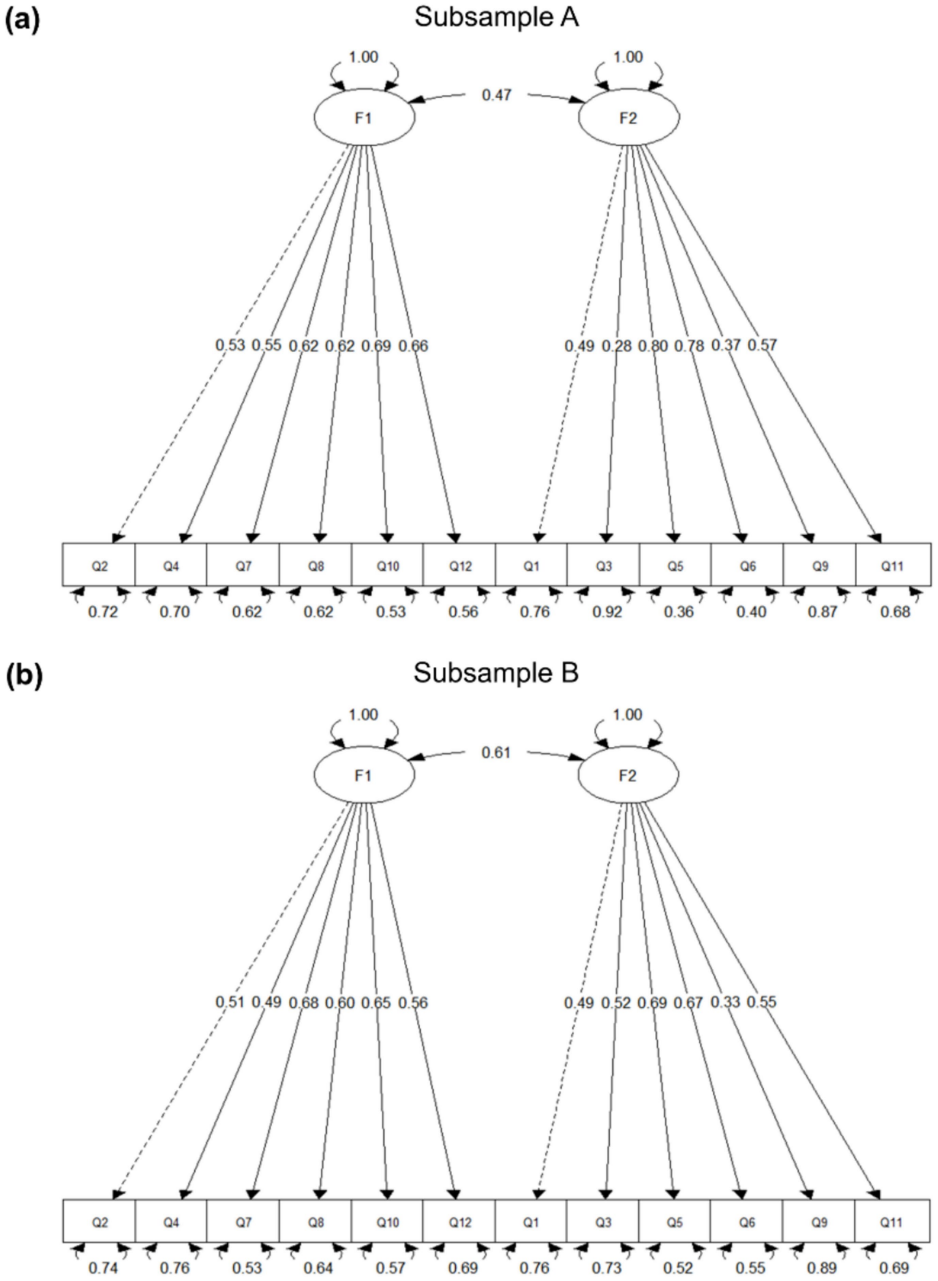
Path diagram of the two-factor CFA model. **(a)** Corresponds to Subsample A; **(b)** corresponds to Subsample B. Standardized factor loadings are displayed along the arrows. In both subsamples, all items loaded strongly on their respective latent factors, with most loadings exceeding 0.50. The correlation between F1 and F2 was 0.47 in Subsample A and 0.61 in Subsample B.

For Sample A, the CFA model demonstrated a good fit: robust *χ*^2^(53) = 94.60, robust comparative fit index (CFI) = 0.956, robust Tucker–Lewis index (TLI) = 0.946, robust root mean square error of approximation (RMSEA) = 0.047, and Standardized Root Mean Square Residual (SRM) = 0.047. All standardized factor loadings were significant (*p* < 0.001), with most exceeding 0.50 (Figure 3). CR values for the two latent factors were 0.76 and 0.73, both exceeding the recommended threshold of 0.70, indicating acceptable internal consistency. Although the CR values for both factors were acceptable (0.76 and 0.73), the AVE values were below the conventional threshold of 0.50 (F1 = 0.35; F2 = 0.33), indicating that convergent validity was not fully supported and should therefore be interpreted with caution. Methodological references such as Hair et al. (2019) and Cheung et al. (2024) suggest that convergent validity should be evaluated with reference to multiple indicators, including factor loadings, composite reliability, and the broader measurement context, rather than on the basis of a single statistic alone. In the present study, all standardized factor loadings were statistically significant and the overall model fit was acceptable. Accordingly, although convergent validity was limited, the two-factor structure may still be considered suitable for preliminary group-level assessment.

For Sample B, the model fit remained acceptable, though slightly lower: robust *χ*^2^(53) = 128.95, robust CFI = 0.923, robust TLI = 0.904, robust RMSEA = 0.059, and SRMR = 0.054. All items loaded significantly onto their respective factors, with loading patterns generally consistent with those in Sample A (Figure 3). CR values remained acceptable (F1 = 0.76; F2 = 0.73), while AVE values fell below the 0.50 threshold (F1 = 0.35; F2 = 0.33). Factor correlations were moderate in both samples (Sample A: *r* = 0.47; Sample B: *r* = 0.61), supporting the discriminant validity of the two-factor structure.

To further assess the potential influence of common method bias, Harman’s single-factor test was conducted. The first factor accounted for 26% of the total variance, which is below the commonly accepted threshold of 40%, suggesting that common method bias is unlikely to be a serious concern (Podsakoff et al., 2003).

**Figure 4:**
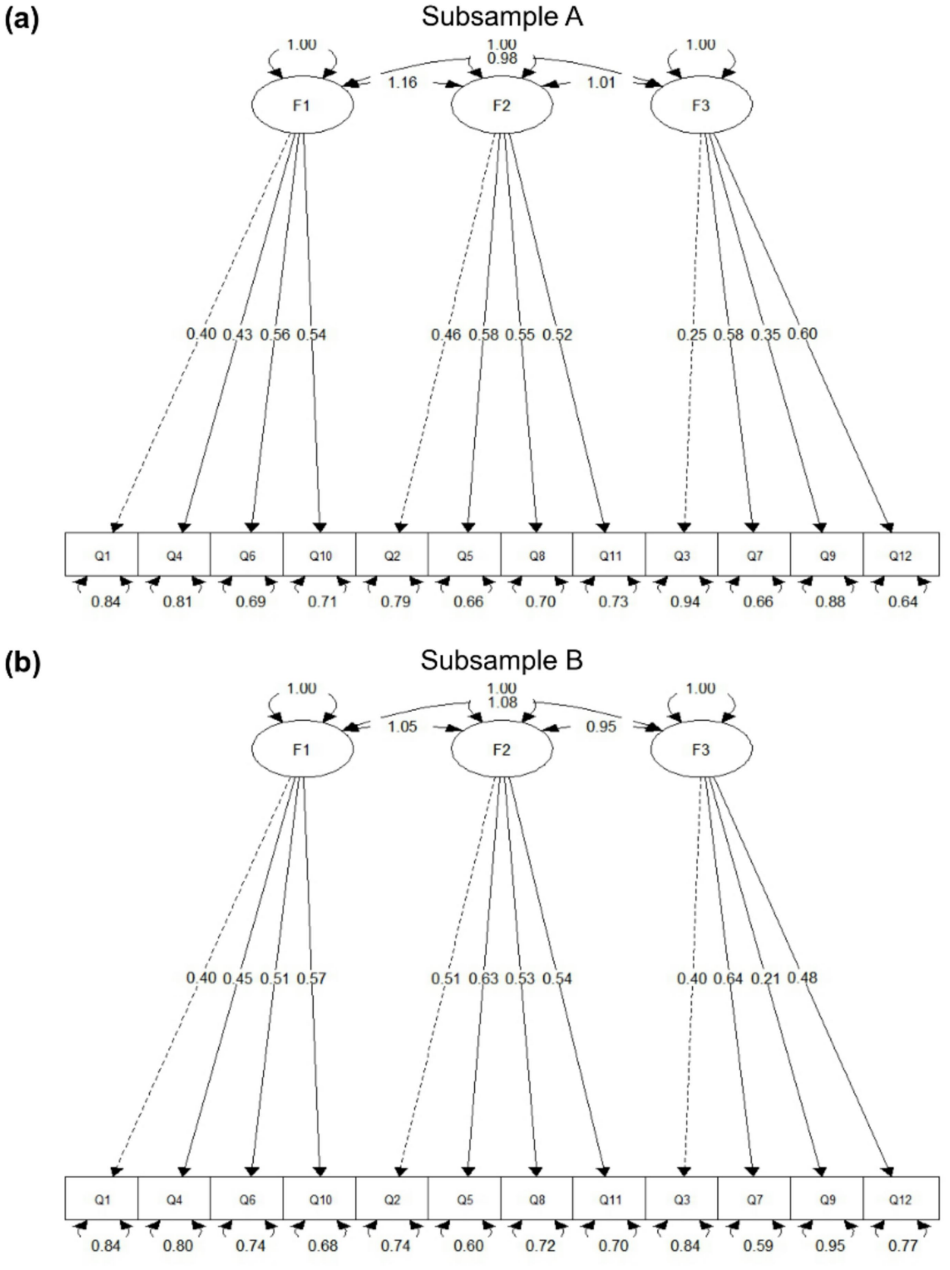
Path diagram of the alternative three-factor CFA model: **(a)** Subsample A; **(b)** Subsample B. Standardized factor loadings are displayed along the arrows. The model showed substantial overlap among the latent factors, with some inter-factor associations approaching or exceeding the admissible boundary, and the latent covariance matrix was not positive definite.

To evaluate the robustness of the factor structure, an alternative three-factor model based on the original theoretical dimensions (cognitive, affective, behavioral) was tested using the English version of the ATTARI-12, including F1 (cognitive: Q1, Q4, Q6, Q10), F2 (affective: Q2, Q5, Q8, Q11), and F3 (behavioral: Q3, Q7, Q9, Q12) components. The results showed that all standardized loadings were significant and the overall model fit was acceptable. The alternative three-factor model exhibited serious estimation problems. Specifically, the latent correlations among the factors were extremely high, with some estimates approaching or exceeding 1.00, and the latent covariance matrix was not positive definite. These findings indicate poor discriminant validity and an inadmissible solution, suggesting that the three hypothesized dimensions were not empirically separable in the present sample (Figure 4). Therefore, the two-factor model was retained for subsequent analysis.

#### Internal consistency reliability

Internal consistency reliability was assessed using the total sample (*n* = 920). Cronbach’s alpha for the overall scale was 0.79, exceeding the commonly accepted threshold of 0.70. McDonald’s omega (*ω*) was satisfactory for the total scale (*ω* = 0.82). Following the identification of the factor structure through EFA, internal consistency was evaluated for each subscale. Cronbach’s alpha was 0.77 for F1 and 0.71 for F2, both indicating acceptable reliability. The hierarchical omega (ωₕ = 0.54) indicated moderate general saturation, supporting a multidimensional structure. Item–total correlations ranged from 0.40 to 0.65, supporting adequate item-level reliability.

#### Invariance test

After establishing the two-factor structure of the ATTARI-12 through EFA and CFA with satisfactory internal consistency reliability, the invariance test across gender and academic discipline was examined using multi-group confirmatory factor analysis (MG-CFA) based on the total sample (*n* = 920). Table 2 presents the results of the tests across genders and academic disciplines.

**Table 2:** Invariance test for the Chinese version ATTARI-12 across gender and academic discipline.

Group	Model	*χ*^2^(df)	CFI	RMSEA	ΔCFI	ΔRMSEA	*p*
Gender	Configural	248.06(106)	0.940	0.054	–	–	–
	Metric	263.13(116)	0.938	0.053	0.002↓	0.001↓	0.129
	Scalar	277.16(126)	0.936	0.051	0.002↓	0.002↓	0.172
Academic discipline	Configural	227.39(106)	0.948	0.050	–	–	–
	Metric	240.48(116)	0.947	0.048	0.001↓	0.002↓	0.219
	Scalar	261.50(126)	0.942	0.048	0.005↓	0.000	0.021*

↓ indicates a decrease in the model fit index compared to the less constrained model. * indicates a statistically significant change in model fit based on the *χ*^2^ difference test at *p* < 0.05.

For the gender groups (male = 395, female = 525), the model showed a good fit at all three levels (configural: CFI = 0.940, RMSEA = 0.054, SRMR = 0.044; scalar: CFI = 0.936, RMSEA = 0.051, SRMR = 0.051). The changes in model fit across nested models were minimal (ΔCFI ≤ 0.002, ΔRMSEA ≤ 0.001, ΔSRMR ≤ 0.006), and chi-square difference tests were non-significant (all *p* > 0.10), supporting full scalar invariance across genders.

For the academic discipline (STEM = 391, HSS = 529), configural and metric invariance were supported (configural: CFI = 0.948, RMSEA = 0.050, SRMR = 0.042; metric: CFI = 0.947, RMSEA = 0.048, SRMR = 0.047). However, the scalar model showed a slight decrease in model fit (scalar: CFI = 0.942, RMSEA = 0.048, SRMR = 0.049), with a marginally significant chi-square difference test (*p* = 0.021). Despite this, changes in CFI (ΔCFI = 0.005) and RMSEA (ΔRMSEA = 0) remained within the recommended thresholds (ΔCFI ≤ 0.01). These results indicate that full scalar invariance was achieved across gender, while partial scalar invariance was supported across academic disciplines, which is generally considered sufficient for meaningful group comparison.

### Descriptive statistics and group comparisons

#### Descriptive statistics

**Figure 5:**
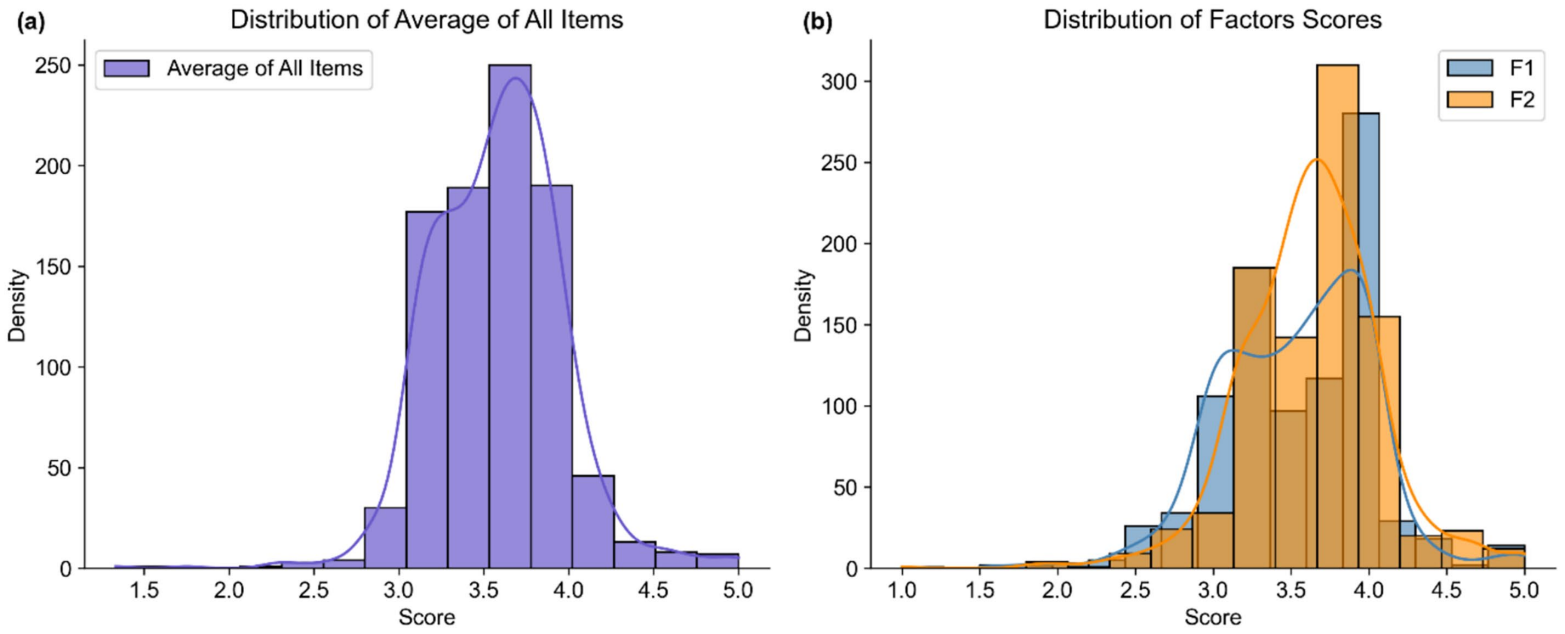
Distribution of average of all items and factor scores. Panel **(a)** shows the distribution of the average score across all 12 items. Panel **(b)** displays the distributions of scores on F1 and F2.

Descriptive statistics for the 12 items and two derived factors of the Chinese version of the ATTARI-12 for students are presented in Table 3. Item-level means ranged from 3.15 (Q9) to 3.96 (Q5), with most items demonstrating moderate to high average scores. Several items (e.g., Q1, Q2, and Q5) exhibited notable negative skewness (skewness < −1), indicating a general tendency toward agreement. F1 had a mean of 3.53 (SD = 0.48), and F2 had a mean of 3.72 (SD = 0.46), both reflecting predominantly favorable attitudes toward AI (Figure 5).

**Table 3:** Descriptive statistics (mean, SD, skewness, kurtosis) of scores for the items, factors and total ATTARI-12 scale (total sample, *n* = 920).

Variable	Mean	SD	Skewness	Kurtosis
Q1	3.80	0.65	−1.04	3.33
Q2	3.67	0.72	−1.00	1.70
Q3	3.44	0.78	−0.54	0.41
Q4	3.41	0.71	−0.46	0.32
Q5	3.96	0.61	−1.03	3.75
Q6	3.80	0.64	−0.86	1.92
Q7	3.46	0.71	−0.21	−0.09
Q8	3.56	0.74	−0.43	0.07
Q9	3.15	0.80	−0.09	−0.21
Q10	3.55	0.68	−0.53	0.54
Q11	3.61	0.67	−0.83	0.97
Q12	3.55	0.71	−0.62	0.33
Factor 1 (Negative Items)	3.53	0.48	−0.20	0.80
Factor 2 (Positive Items)	3.63	0.44	−0.30	2.55
Average of All Items	3.58	0.73	−0.63	0.63

F1 consists of Q2, Q4, Q7, Q8, Q10, Q12; F2 consists of Q1, Q3, Q5, Q6, Q9, Q11; Average of all Items represents the average score across all items.

**Figure 6:**
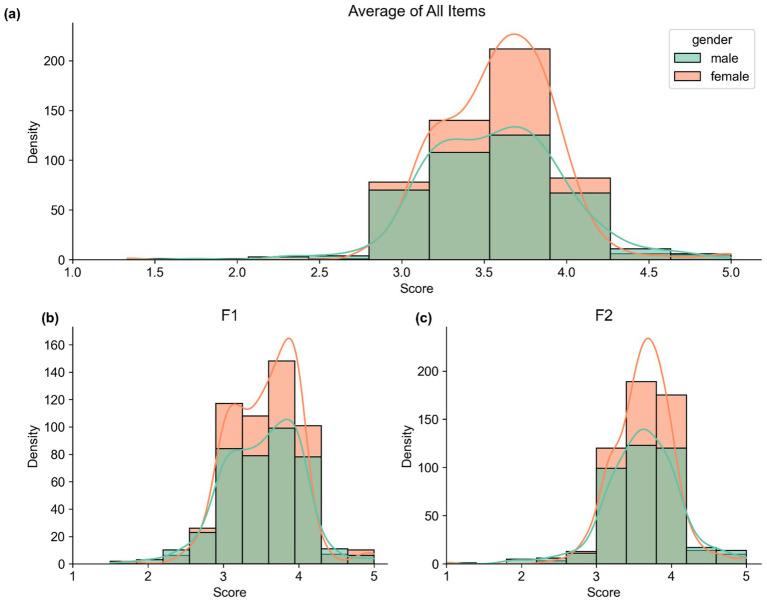
Distribution of scores by gender. Panel **(a)** shows the distribution of the average score across all 12 items of the ATTARI-12 scale. Panel **(b)** displays the distribution of scores on the F1 scale, which comprises negatively worded items. Panel **(c)** presents the distribution of scores on F2, which includes positively worded items.

**Figure 7:**
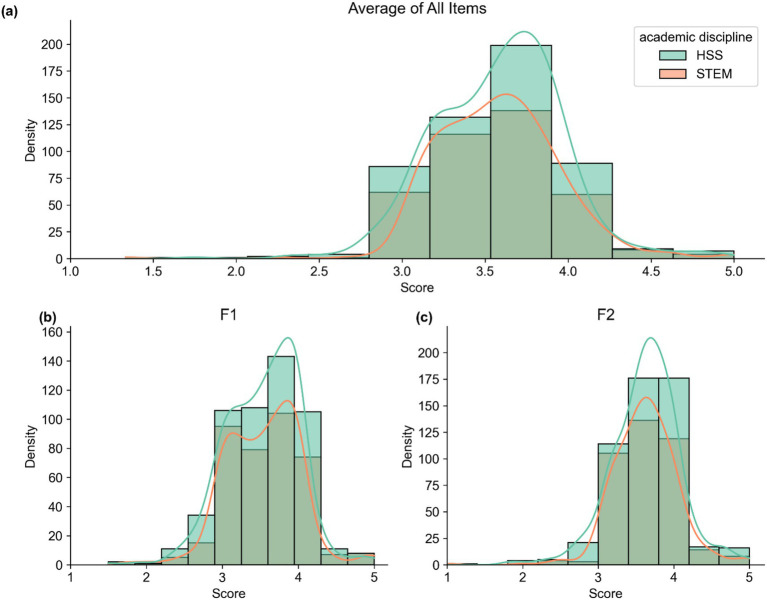
Distribution of scores by academic discipline. Panel **(a)** shows the distribution of the average score across all 12 items of the ATTARI-12 scale. Panel **(b)** displays the distribution of scores on F1, which comprises negatively worded items. Panel **(c)** presents the distribution of scores on F2, which includes positively worded items.

Female students reported relatively consistent scores for both F1 (Mean = 3.55, SD = 0.46) and F2 (Mean = 3.63, SD = 0.40), whereas male students showed similar distributions for F1 (Mean = 3.52, SD = 0.51) and F2 (Mean = 3.62, SD = 0.49). In terms of academic discipline, students from HSS reported F1 = 3.54 (SD = 0.48) and F2 = 3.62 (SD = 0.43), whereas STEM students reported F1 = 3.53 (SD = 0.48) and F2 = 3.63 (SD = 0.45) (see Table 4 and Figures 6, 7). Therefore, these scores indicate a consistent pattern of positive attitudes toward AI across the subgroups.

**Table 4:** Descriptive statistics of scores by gender and academic discipline.

Group	Variable	Mean	SD	Skewness	Kurtosis
Gender: Male	F1	3.52	0.51	−0.31	0.66
	F2	3.62	0.49	−0.25	1.40
	Average of All Items	3.57	0.77	−0.59	0.53
Gender: Female	F1	3.55	0.46	−0.06	0.81
	F2	3.63	0.40	−0.34	3.86
	Average of All Items	3.59	0.70	−0.65	0.65
Academic discipline: HSS	F1	3.54	0.48	−0.08	1.03
	F2	3.62	0.43	−0.35	4.12
	Average of All Items	3.58	0.73	−0.58	0.58
Academic discipline: STEM	F1	3.53	0.48	−0.28	0.62
	F2	3.63	0.45	−0.27	1.54
	Average of All Items	3.58	0.73	−0.67	0.66

#### Group comparisons

A 2 × 2 factorial analysis of variance (ANOVA) was conducted on F1 scores, revealing no significant main effects of gender [*F*(1, 916) = 1.01, *p* = 0.314] or academic discipline [*F*(1, 916) = 0.03, *p* = 0.870]. The interaction was also not significant, *F*(1, 916) = 0.15, *p* = 0.699 (Table 5).

**Table 5:** Results of normality, homogeneity, and group comparison tests for factors and average of all items.

Variable	Shapiro–Wilk normality (p)^1^	Levene’s test for homogeneity (p)^2^	Test method	Gender effect (p)	Background effect (p)	Interaction (p)
F1 (Negative Items)	0.001*	0.202	Two-way ANOVA	0.314	0.870	0.699
F2 (Positive Items)	0.001*	0.001*	Scheirer–Ray–Hare test	0.942	0.477	0.728
Average of All Items	0.001*	0.001*	Scheirer–Ray–Hare test	0.380	0.592	0.862

Normality test results are summarized based on all four groups (gender × academic discipline). All *p*-values were less than 0.01, indicating a significant deviation from normality. The Levene’s test was used to assess the homogeneity of variances across the 2 × 2 design. Significant values (*p* < 0.01) indicate heterogeneity. Significance codes: *p < 0.001.

For F2 and the average of all item scores, the Scheirer–Ray–Hare nonparametric two-way tests were employed because of violations of normality and homogeneity of variance. No significant effects were observed for either variable in terms of gender, academic discipline, or their interaction (all *p* > 0.05) (Table 5).

## Discussion

### Validity and structure of the Chinese ATTARI-12

The findings of this study suggest that the Chinese version of the ATTARI-12 has satisfactory content and construct validity. The expert-based content validity assessment yielded consistently high I-CVI and S-CVI scores, thus supporting the clarity and relevance of the translated items.

Regarding construct validity, the EFA supported a two-factor model corresponding to positive and negative items. This structure diverges from the original three-factor model, which is based on functional dimensions: cognitive, affective, and behavioral. Therefore, a three-factor model, aligned with the original ATTARI-12, was tested. Although the three-factor model met acceptable fit criteria, the extremely high correlations among the latent variables (*r* > 0.98) and a non-positive-definite covariance matrix indicated poor discriminant validity and structural instability (Figure 4) (Pan et al., 2017; Brown, 2015). Consequently, the two-factor model is retained. The two-factor model may reflect a broader cognitive tendency to organize attitudinal responses along emotional valence (positive vs. negative) rather than abstract conceptual dimensions. This interpretation is also consistent with psychometric research suggesting that when theoretically distinct components are highly correlated, more parsimonious factor structures may better represent the underlying construct (Clark and Watson, 1995). In our study, the extremely high correlations observed in the three-factor model (*r* > 0.98) indicate a lack of discriminant validity, thereby supporting the adoption of a more parsimonious two-factor solution. In fact, previous research has supported this interpretation. For example, Briesemeister et al. (2012) found that emotionally ambivalent words are processed more efficiently than neutral ones, indicating a preference for affective processing over semantic processing. Similarly, Rocklage and Fazio (2016) showed that emotionally intense components often dominate the overall evaluations when individuals are exposed to mixed-valence information. In the present context, this may suggest that Chinese undergraduates form attitudes toward AI primarily on the basis of emotional impressions rather than distinct cognitive, affective, or behavioral dimensions. The increasing integration of AI into daily life and education may reinforce this effect-driven structure. Moreover, given that the respondents were undergraduate students with limited direct experience in AI development, their attitudes are likely to be shaped by general evaluative polarity rather than detailed conceptual elaboration. The present findings extend beyond a purely statistical validation of the scale. The emergence of a two-factor structure based on emotional valence (positive vs. negative attitudes) suggests that undergraduate students may organize their perceptions of AI primarily along affective dimensions rather than distinct cognitive, affective, and behavioral components. This valence-driven structure is consistent with recent findings in diverse educational settings (Nurtanto et al., 2025; Hamkah et al., 2025), where students’ behavioral intentions are strongly mediated by their initial affective predispositions toward AI tools.

All 12 items from the original scale were retained in the CFA, including Q9 (*I would rather choose a technology with AI than one without it*). Although Q9 demonstrated comparatively lower factor loading and central tendency, it captures specific behavioral preferences relevant to AI use in everyday contexts (Stein et al., 2024). Its inclusion was considered valuable for maintaining conceptual coverage and alignment with the original scale (Cheung et al., 2024; Boateng et al., 2018). The relatively lower response average on Q9 may not reflect weaker endorsement but rather uncertainty or limited practical familiarity with AI-embedded technologies. Furthermore, the item’s wording blends attitude preference and behavioral choice, which may partially account for the lower loading.

In terms of convergent validity, both factors achieved CR values greater than the recommended threshold of 0.70. Although the AVE was slightly below 0.50, the strong item loadings and satisfactory composite reliability supported the instrument’s adequacy for practical use (Jain et al., 2025). These results suggest that, while some fit indices in Subsample B (CFI = 0.923) were slightly below the more stringent recommended thresholds (CFI ≥ 0.95), the overall model fit remained acceptable when considered alongside theoretical interpretability, consistent factor loadings, and model parsimony. The marginally insufficient AVE may stem from the concise item set and the relatively narrow semantic range within each factor, which limit the total variance explained. Similarly, while the two retained factors explained approximately 35% of the total variance, which is within the acceptable range for psychological constructs, this figure remains at the lower end of desirable thresholds (Beierlein et al., 2016). This suggests that the additional meaningful variance may not be fully captured in the current configuration.

Internal consistency was acceptable, with Cronbach’s alpha (*α* = 0.79) and McDonald’s omega (*ω* = 0.82) exceeding the conventional benchmarks for both the overall scale and its subscales. The invariance test confirmed that the two-factor structure was equivalent across gender and academic discipline, indicating stable structural properties across the subgroups. The establishment of full scalar invariance across gender indicates that latent mean comparisons between male and female students are valid. This finding indicates that comparisons of latent means between male and female students are meaningful and not biased by measurement differences. Therefore, any observed similarities or differences in AI attitudes across gender can be interpreted as reflecting true attitudinal patterns rather than artifacts of measurement.

For academic disciplines, while configural and metric invariance were supported, the scalar model showed a slight decrement in fit, and the chi-square difference test was marginally significant (*p* = 0.021). However, the changes in CFI and RMSEA remained within acceptable thresholds (ΔCFI ≤ 0.01), supporting partial scalar invariance (Lin et al., 2017; Mak et al., 2020). This level of invariance is generally considered sufficient to allow cautious comparisons of latent constructs across groups. These findings suggest that the ATTARI-12 operates in a largely equivalent manner across STEM and HSS students, supporting its use in cross-group comparisons.

Therefore, the findings of validity and structure provide evidence that the Chinese version of the ATTARI-12 demonstrates acceptable levels of validity and structural stability for assessing attitudes toward AI among university students in China.

### Attitudinal patterns and group comparisons

Descriptive statistics indicated that Chinese undergraduates generally held positive attitudes toward AI, with most item scores falling within the moderate to high range and exhibiting negative skewness, suggesting a tendency toward agreement (Table 3). For example, items such as Q1 (*AI will make this world a better place*) and Q5 (*I look forward to future AI developments*) received particularly high mean scores, reflecting widespread endorsement. These findings align with those of previous studies conducted across different cultural contexts. For instance, university students in India demonstrated similar positive attitudes, with science majors reporting more favorable views than their peers in arts and commerce (Hajam and Gahir, 2024). In the United Kingdom, Otermans et al. (2025) found that students’ cognitive and behavioral attitudes were positively associated with their awareness and use of AI in education. Greek students also expressed moderately positive attitudes, with strong support for ethical AI use, and a positive correlation between digital literacy and AI acceptance (Saklaki and Gardikiotis, 2024). Similarly, Tien (2024) reported that Vietnamese undergraduates generally held positive views of AI, with no significant gender differences, although attitudes varied by year of study.

In our study, no significant differences were found across genders or academic disciplines, suggesting a relatively uniform attitudinal pattern among Chinese undergraduates. Our results are consistent with those of studies from Vietnam and India, where demographic factors such as gender often cease to predict AI attitudes once educational or technological access is accounted for Tien (2024) and Hajam and Gahir (2024). However, other studies have also suggested that such convergence is not universal. For example, male students in technical fields in Austria and Serbia expressed significantly more positive attitudes toward AI compared to other groups (Adžić et al., 2024), and similar disciplinary differences were observed among Turkish EFL learners, where engineering and science students showed greater enthusiasm for AI integration in learning (Korkmaz and Akbıyık, 2024).

These cross-cultural findings suggest that, while student attitudes toward AI may converge in some educational contexts, significant differences across academic disciplines and gender persist in other contexts. This indicates that patterns of student attitudes toward AI remain shaped by contextual and cultural factors and uniformity should not be assumed across all settings.

### Limitations and future directions

This study had several limitations that warrant consideration. First, although the CR values and standardized factor loadings were acceptable, the AVE values for both factors fell below the conventional threshold, indicating that convergent validity was limited. This suggests that some items may not have captured the latent constructs with optimal precision. Given the brevity of the scale and the relatively narrow semantic range of the retained items, future research should consider modest item expansion, wording refinement, or increased semantic diversity to strengthen convergent validity. In addition, further psychometric evidence, such as test–retest reliability or temporal stability, would be valuable, particularly because internal consistency alone should not be treated as a substitute for retest reliability when evaluating scale quality (McCrae et al., 2011).

Furthermore, the two-factor model accounted for approximately 35% of the total variance, which, while acceptable for attitudinal constructs, suggests that additional meaningful variances may remain unmeasured. Future studies might consider incorporating advanced analytical techniques, such as semantic network analysis and natural language processing, to better capture the cognitive and cultural structures of attitudes toward AI.

Finally, the sample was drawn from a single university in southwestern China, which may constrain the geographic representation of the findings. While the study specifically targeted undergraduate students, concentration within one institution limits the generalisability of the results across diverse educational contexts and regions within China’s broader higher education landscape. Future research should replicate the validation process across a broader range of institutions and student populations to enhance the scale’s external validity. Critically, AI attitudes are embedded within specific socio-technical contexts; in China, the national strategic emphasis on AI might foster a general ‘algorithm appreciation’ that could vary across different tiers of universities (Logg et al., 2019). This institutional concentration may have influenced the emergence of the two-factor valence structure observed here. Future research should employ multi-site sampling to test the scalar invariance of the ATTARI-12 across diverse institutional environments and to account for varying levels of AI exposure.

## Conclusion

This study validated the Chinese version of the ATTARI-12 scale for assessing attitudes toward AI among undergraduate students. The two-factor structure based on emotional valence showed acceptable reliability, model fit, and measurement invariance across gender and academic disciplines, supporting the scale’s applicability in the Chinese higher education context.

The results also indicate that Chinese students in the present sample have positive attitudes toward AI, as reflected in the high item scores. This pattern aligns with the trends observed in other countries. However, findings on the influence of academic discipline and gender remain mixed, with studies reporting inconsistent patterns in students’ attitudes toward AI. Given the context-specific nature of the sample, further validation across diverse cultural and educational settings is warranted to establish broader generalizability.

## Data Availability

The raw data supporting the conclusions of this article will be made available by the authors, without undue reservation.

## Footnotes

1
https://doi.org/10.1038/s41598-024-53335-2

2
https://www.wjx.cn/
