# Supplementary material for: Measuring attitudes toward artificial intelligence: validation of the Chinese ATTARI-12 and invariance across genders and academic disciplines
Source: Front Psychol. 2026 May 15;17:1836060. doi: 10.3389/fpsyg.2026.1836060 (PMC13219287; doi:10.3389/fpsyg.2026.1836060)
Supplement: Supplementary file 1 [file Data_Sheet_1.PDF]

| Item | Chinese | English | Item Polarity |
| --- | --- | --- | --- |
| Q1 | 人工智能将使这个世界变得更美好。 | AI will make this world a better place. | Positive |
| Q2 | 我对人工智能有强烈的负面情绪。 | I have strong negative emotions about AI. | Negative |
| Q3 | 我想使用依赖于人工智能的技术。 | I want to use technologies that rely on AI. | Positive |
| Q4 | 人工智能的缺点多于优点。 | AI has more disadvantages than advantages. | Negative |
| Q5 | 我期待未来人工智能的发展。 | I look forward to future AI developments. | Positive |
| Q6 | 人工智能为许多世界性问题提供了解决方案。 | AI offers solutions to many world problems. | Positive |
| Q7 | 我更喜欢不含人工智能的技术。 | I prefer technologies that do not feature AI. | Negative |
| Q8 | 我害怕人工智能。 | I am afraid of AI. | Negative |
| Q9 | 我宁愿选择带有人工智能的技术，而不是没有人工智能的技术。 | I would rather choose a technology with AI than one without it. | Positive |
| Q10 | 人工智能制造问题，而不是解决问题。 | AI creates problems rather than solving them. | Negative |
| Q11 | 当我想到人工智能时，我大多有正面的感受。 | When I think about AI, I have mostly positive feelings. | Positive |
| Q12 | 我宁愿避免使用基于人工智能的技术。 | I would rather avoid technologies that are based on AI. | Negative |

**Supplementary Table S1**. The bilingual items (Chinese and English) of the ATTARI-12.
